# Spinal Hb9::Cre-derived excitatory interneurons contribute to rhythm generation in the mouse

**DOI:** 10.1038/srep41369

**Published:** 2017-01-27

**Authors:** Vanessa Caldeira, Kimberly J. Dougherty, Lotta Borgius, Ole Kiehn

**Affiliations:** 1 Mammalian Locomotor Laboratory, Department of Neuroscience, Karolinska Institutet, 171 77 Stockholm, Sweden; 2 Department of Neurobiology and Anatomy, Drexel University College of Medicine, Philadelphia, PA 19129, US

## Abstract

Rhythm generating neurons are thought to be ipsilaterally-projecting excitatory neurons in the thoracolumbar mammalian spinal cord. Recently, a subset of Shox2 interneurons (Shox2 non-V2a INs) was found to fulfill these criteria and make up a fraction of the rhythm-generating population. Here we use *Hb9*::*Cre* mice to genetically manipulate Hb9::Cre-derived excitatory interneurons (INs) in order to determine the role of these INs in rhythm generation. We demonstrate that this line captures a consistent population of spinal INs which is mixed with respect to neurotransmitter phenotype and progenitor domain, but does not overlap with the Shox2 non-V2a population. We also show that Hb9::Cre-derived INs include the comparatively small medial population of INs which continues to express Hb9 postnatally. When excitatory neurotransmission is selectively blocked by deleting Vglut2 from Hb9::Cre-derived INs, there is no difference in left-right and/or flexor-extensor phasing between these cords and controls, suggesting that excitatory Hb9::Cre-derived INs do not affect pattern generation. In contrast, the frequencies of locomotor activity are significantly lower in cords from *Hb9*::*Cre-Vglut2*^Δ*/*Δ^ mice than in cords from controls. Collectively, our findings indicate that excitatory Hb9::Cre-derived INs constitute a distinct population of neurons that participates in the rhythm generating kernel for spinal locomotion.

Locomotor behaviors, such as flying, swimming or walking, are complex motor actions that give animals and humans the ability to move and interact with the surroundings. The generation of locomotion in vertebrates is largely determined by neural networks in the spinal cord^1^,^2^,^3^,^4^. These spinal networks are responsible for key features that characterize limbed locomotion in mammals, rhythm generation and the precise coordination of muscle activation. In the ventral spinal cord where the locomotor circuit is located^5^, developmentally expressed transcription factors^6^,^7^ have allowed for the identification and targeted manipulation of selected interneuron populations^3^,^8^,^9^,^10^. These studies have provided insights into the organization of the neuronal circuits underlying left-right^11^,^12^,^13^,^14^,^15^,^16^ and flexor-extensor^17^,^18^,^19^ coordination in mammals. Optogenetic, pharmacological, and lesion studies have provided strong evidence that the rhythmic drive in the mammalian spinal locomotor circuit comes from activity in ipsilaterally-projecting excitatory glutamatergic neurons^1^,^2^,^3^,^9^,^20^,^21^,^22^,^23^,^24^. However, despite the ablation of entire progenitor domain-derived classes of ipsilaterally-projecting excitatory neurons, rhythm-generating interneurons still remain elusive^12^,^14^,^16^,^25^,^26^,^27^. Therefore, there has also been focus on identifying and studying cell populations that span several progenitor domains. Recently, one such study directly linked rhythm generation to a group of excitatory interneurons identified by the embryonic expression of the transcription factor short stature homeobox protein 2 (Shox2)^28^. Shox2 neurons span several of the dorsally and ventrally progenitor domain-derived interneuron classes, and partially overlap with V2a neurons which express the transcription factor Chx10 during development^28^,^29^,^30^. Shox2 non-V2a neurons were shown to be involved in rhythm generation^28^. However, eliminating Shox2 non-V2a neurons from the network did not result in complete abolition of locomotor rhythm. Collectively, these data suggest that glutamatergic interneuron populations other than those captured in the current transcription factor schemas are involved in rhythm generation. To target such populations we are examining currently available genetic tools that may define unique neuronal groups to which we can ascribe such a function.

One group of interest is the group of neurons labeled by the transcription factor Hb9. Hb9 is usually described as a postmitotic motor neuron marker^31^, however, it is also transiently expressed in a larger group of progenitor cells and postmitotic neurons embryonically^32^. As it is commonly done when targeting spinal cord interneuron populations, we use conditional genetics and rely on Cre-dependent recombination to reliably target excitatory interneurons identified by the developmental expression of Hb9. This approach includes the small medial subset of interneurons retaining HB9 protein expression postnatally, which have been extensively studied and found to be excitatory neurons displaying cellular properties consistent with being potential rhythm generating neurons^33^,^34^,^35^,^36^,^37^,^38^,^39^.

In the current study, we selectively silence glutamatergic synaptic transmission in Hb9::Cre-derived excitatory neurons while retaining motor neuron output. We demonstrate that although reporter expression observed in *Hb9*::*Cre* mice represents a much larger population of neurons than those expressing the HB9 protein postnatally, the population identified is consistent and can be reliably used to target and manipulate a novel excitatory neuronal population in the spinal cord. We also show that excitatory Hb9::Cre-derived interneurons do not overlap with the Shox2 non-V2a population. Synaptically silencing the excitatory subset of Hb9::Cre-derived interneurons by a targeted deletion of the vesicular glutamate transporter 2 (Vglut2) leads to a significant reduction in locomotor frequency without any significant effect in pattern formation, suggesting a role in rhythm generation. Taken together, our findings indicate that excitatory Hb9::Cre-derived interneurons constitute a second population of neurons, distinct from the Shox2 non-V2a, which appear to be involved in the rhythm-generating kernel for mammalian locomotion.

## Results

### Hb9::Cre-derived INs are located in the ventral and dorsal spinal cord

Although *Hb9*::*Cre*^40^ and *Hb9-GFP*^41^ mice were generated with an analogous strategy to the *Hb9-LacZ* mice^31^, reporter expression in *Hb9*::*Cre-reporter-labeled (Hb9*::*Cre;Rosa26-FP*) and *Hb9-GFP* mice is not restricted to motor neurons (MNs). In the *Hb9-GFP* mouse, a ventral population of interneurons is marked^41^, whereas in the *Hb9*::*Cre;Rosa26-FP* mouse line a dorsal population of cells is also captured. The increase in the number and laminar distribution of fluorescent reporter cells observed in *Hb9*::*Cre;Rosa26-FP* mice may be attributed to the transient embryonic expression of Hb9 in these cells^32^,^42^.

**Figure 1:**
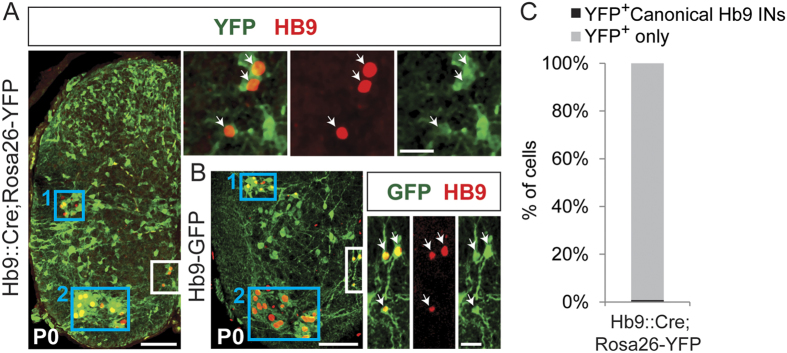
Distribution of Hb9::Cre-derived INs and canonical Hb9 INs in the mouse rostral lumbar P0 spinal cord. (**A**) Distribution of Hb9::Cre-derived INs (green), as marked by YFP expression (*Hb9*::*Cre;Rosa26-YFP* mice), and canonical Hb9 INs (white boxed area), as marked by HB9 protein expression (red) in medial lamina VIII. Preganglionic neurons (blue box 1) and motor neurons (blue box 2) also express HB9 protein in both *Hb9-GFP* and *Hb9*::*Cre; Rosa26-YFP* mice. Upper rightmost pictures are magnifications of the white boxed area containing canonical Hb9 INs. Arrowheads indicate overlap between Hb9::Cre-derived INs (YFP, green) and canonical Hb9 INs (Hb9 antibody, red). Scale bars: 100 μm and 25 μm. (**B**) Distribution of eGFP neurons (green) in the ventral spinal cord of *Hb9-GFP* mice, and the subset of canonical Hb9 INs (white boxed area), as marked by the overlap of HB9 protein (red) and GFP expression in medial lamina VIII. Rightmost pictures are magnifications of the white boxed area. Arrowheads indicate overlap between GFP (green) and HB9 protein (red). Scale bars: 100 μm and 25 μm. (**C**) Bar graph showing the percentage of the Hb9::Cre-derived IN population (represented by YFP expression, YFP^+^) that corresponds to canonical Hb9 INs (YFP^+^ Canonical Hb9 INs) in *Hb9*::*Cre;Rosa26-YFP* mice. Canonical Hb9 INs account for less than 1% (0.86% ± 0.37%, darker grey) of the Hb9::Cre-derived IN population.

YFP expression in *Hb9*::*Cre;Rosa26-YFP* mice was detected through lamina I to VI in addition to lamina VII, VIII and ventral X (Fig. 1A) throughout the lumbar spinal cord. This is in contrast to the GFP expression in *Hb9-GFP* mice^33^, which is restricted to lamina VII, VIII and ventral X (Fig. 1B). We will collectively refer to these dorsal (lamina I-VI) and ventral neuronal populations in *Hb9*::*Cre;Rosa26-FP* mice as Hb9::Cre-derived INs.

Despite the larger number of reporter-expressing cells in *Hb9*::*Cre;Rosa26-FP* mice, reporter expression identifies a consistent group of interneurons throughout the lumbar spinal cord. Thus, we use the *Hb9*::*Cre* mouse as a genetic tool to study the possible roles of excitatory Hb9::Cre-derived INs in locomotion.

### Canonical Hb9 INs account for less than 1% of the Hb9::Cre-derived IN population

We first asked if the Hb9::Cre-derived INs include the canonical Hb9 INs. We define canonical Hb9 INs as the small subset of neurons clustered in medial lamina VIII in the lower thoracic and upper lumbar mouse spinal cord. These interneurons retain endogenous HB9 protein expression postnatally and also co-express GFP protein under the Hb9 promoter in *Hb9-GFP* mice (type I cells referred in refs 33 and 35) (Fig. 1B, white boxed area). These canonical Hb9 neurons have been suggested to be part of the kernel for rhythm generation in the mammalian locomotor network^33^,^34^,^35^,^36^.

In *Hb9*::*Cre;Rosa26-YFP* mice, an overlap of YFP and HB9 protein was evident in canonical Hb9 INs, motor neurons (MNs) and sympathetic preganglionic neurons (Fig. 1A). We indeed found that the majority of canonical Hb9 INs co-express HB9 protein and the reporter protein, YFP, in *Hb9*::*Cre;Rosa26-YFP* mice (86% ± 7%, N = 3, 18 sections). Conversely, canonical Hb9 INs make up less than 1% (0.86% ± 0.37%) of the Hb9::Cre-derived IN population (N = 3, 18 sections) (Fig. 1C). The number of canonical Hb9 neurons (193 ± 61 cells between Th12 and L3, N = 3) is similar to what was previously reported^33^. Taken together these data indicate that most of the canonical Hb9 INs are part of Hb9::Cre-derived IN population, but they account for a very small percentage of this population. Therefore, hereinafter whenever we refer to the Hb9::Cre-derived INs, the canonical Hb9 INs in Th12-L3 will be implicitly included.

### One third of Hb9::Cre-derived INs are glutamatergic

**Figure 2:**
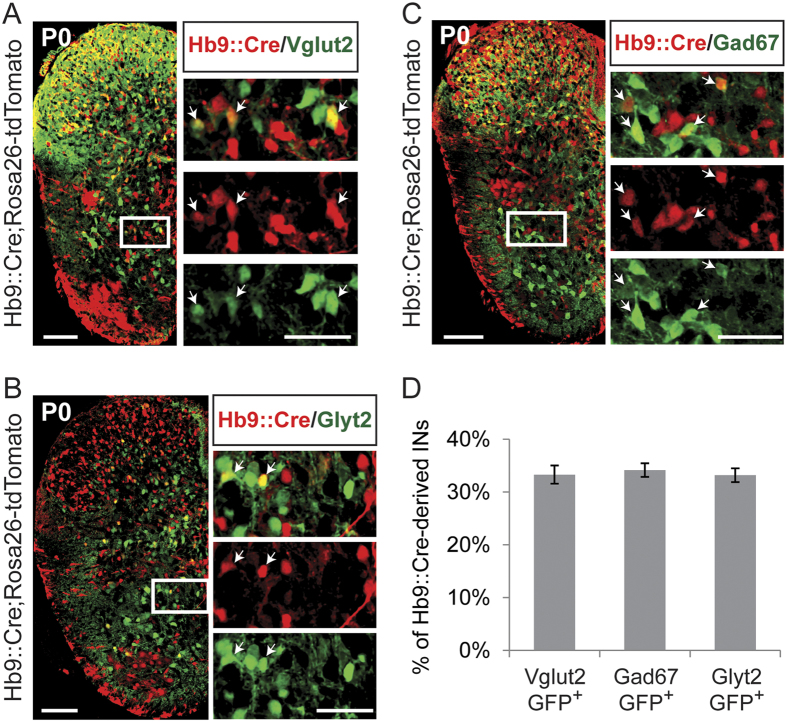
One third of the Hb9::Cre-derived INs are excitatory. (**A**,**B** and **C**) Transverse spinal cord hemi-sections, at segmental levels L1-L3 of P0 mice, showing co-expression of tdTomato fluorescent protein (red) (*Hb9*::*Cre;Rosa26-tdTomato* mice) with GFP (green) from *Vglut2-GFP* (A), *Glyt2-GFP* (B), and *Gad67-GFP* (**C**) mice. Rightmost pictures are magnifications of the white boxed areas. Arrowheads indicate overlap between Hb9::Cre-derived INs (tdTomato protein, red) and Vglut2, Glyt2, or Gad67-GFP neurons (GFP protein, green). Scale bars represent 100 μm for the transverse hemi-sections and 50 μm for the magnified boxes on the right. (**D**) Percent of Hb9::Cre-derived INs that are glutamatergic (Vglut2-GFP^+^, 33% ± 2%), GABAergic (Gad67-GFP^+^, 34% ± 1%), and glycinergic (Glyt2-GFP^+^, 33% ± 1%) in Hb9::Cre;Rosa26-tdTomato spinal cords.

We next sought to assess the transmitter phenotype of the Hb9::Cre-derived IN population by examining overlap of reporter expression in *Hb9*::*Cre;Rosa26-tdTomato;Vglut2-GFP, Hb9*::*Cre;Rosa26-tdTomato;Glyt2-GFP, and Hb9*::*Cre;Rosa26-tdTomato;Gad67-GFP* mice, in order to identify Hb9::Cre-derived glutamatergic (Fig. 2A), glycinergic (Fig. 2B), and GABAergic (Fig. 2C) neurons, respectively. Motor neurons were easily recognized by size and location and were excluded from all counts. The transmitter phenotype of Hb9::Cre-derived INs is mixed; approximately 1/3 are excitatory (33% ± 2% Vglut2) and 2/3 are inhibitory (34% ± 1% GAD67, and 33% ± 1% GlyT2) (N = 3 animals per condition, 48 sections) (Fig. 2D).

### Hb9::Cre-derived INs span several progenitor domains, but constitute a population distinct from the Shox2-nonV2a INs

**Figure 3:**
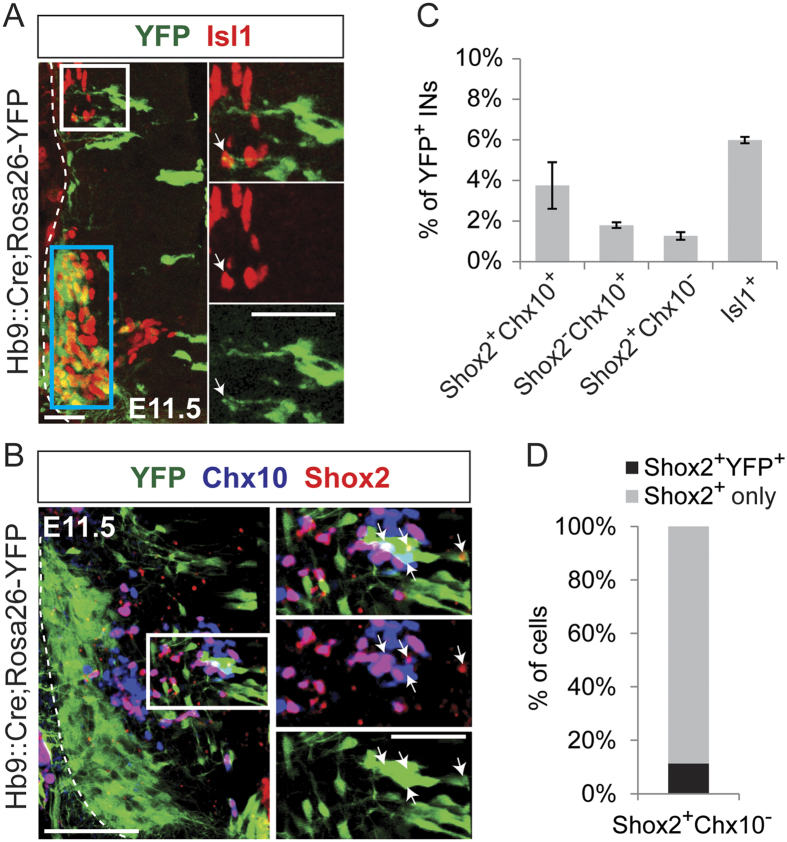
Hb9::Cre-derived INs do not overlap with the Shox2 non-V2a population. (**A**) Co-expression of YFP (green) and Isl1 antibody (red) in the *Hb9*::*Cre;Rosa26-YFP* mouse spinal cord at E11.5. Motor neurons are also labeled by Isl1 antibody (blue box). Rightmost pictures are magnifications of the white boxed area. Arrowheads indicate overlap between Isl1 (red) and Hb9::Cre-derived INs (green). Scale bars: 100 μm and 50 μm. (**B**) Co-expression of YFP (green), Shox2 antibody (red) and/or Chx10 antibody (blue) in the *Hb9*::*Cre;Rosa26-YFP* mouse ventral spinal cord at E11.5. Rightmost pictures are magnifications of the white boxed area. Arrowheads indicate overlap between Hb9::Cre-derived INs (green) and Shox2^+^ Chx10^−^ (red), Shox2^−^ Chx10^+^ (blue) or Shox2^+^ Chx10^+^ (pink). Scale bars: 100 μm and 50 μm. (**C**) Quantification of overlap in (A) and (B). Bar graph showing percent of overlap between Hb9::Cre-derived INs (YFP^+^) and Shox2 V2a (Shox2^+^ Chx10^+^, 4% ± 1%), Shox2^OFF^ V2a (Shox2^−^ Chx10^+^, 2% ± 0.1%), Shox2 non-V2a (Shox2^+^ Chx10^−^, 1.3% ± 0.2%), and Isl1 (Isl1^+^, 6% ± 0.2%) INs. Error bars represent ± SEM. (**D**) Percent of the Shox2 non-V2a IN population (Shox2^+^ Chx10^−^) that overlaps with Hb9::Cre-derived INs (YFP^+^) in the *Hb9*::*Cre;Rosa26-YFP* mouse spinal cord at E11.5. Shox2 non-V2a INs rarely co-express YFP (Shox2^+^ YFP^+^, darker grey) (12% ± 2%). Error bars represent ± SEM.

To further characterize the excitatory Hb9::Cre-derived IN population, we turned to the developmentally expressed transcription factors found in excitatory neurons, and looked for overlap with the exclusively excitatory and ipsilateral IN markers, *Isl1, Chx10* and *Shox2* (Fig. 3A and B respectively). All experiments were performed at embryonic stage E11.5 to capture the peak of expression of these early neuronal markers.

The LIM homeodomain transcription factor 1 (*Isl1*) is expressed in MNs and is involved in MN differentiation^31^. Its expression defines a class of interneurons, the dI3 INs^43^,^44^, which have been shown to be involved in the cutaneous regulation of paw grasp^26^ but not in locomotor rhythm or patterning. Therefore, we sought to investigate whether or not dI3 INs and Hb9::Cre-derived INs were overlapping. We found that Hb9::Cre-derived INs seldomly co-expressed Isl1 (6% ± 0.2%, N = 2, 22 sections) (Fig. 3C), indicating that the dI3 IN population shows no appreciable overlap with the Hb9::Cre-derived INs.

*Chx10* is the marker for V2a neurons and the expression of *Shox2* and *Chx10* can be used to categorize excitatory neurons in the ventral spinal cord into three groups: Shox2 V2a INs (Shox2^+^ Chx10^+^), Shox2 non-V2a INs (Shox2^+^ Chx10^−^), and Shox2^OFF^ V2a INs (Shox2^−^Chx10^+^)^28^. Shox2 non-V2a INs is the only subgroup of excitatory neurons that has been shown to contribute to rhythm generation in mammals, whereas Shox2 V2a INs, and Shox2^OFF^ V2a INs seem to be involved in pattern generation^28^. We found that Hb9::Cre-derived INs rarely overlapped with the Shox2 V2a (4% ± 0.1%), Shox2^OFF^ V2a (2% ± 0.1%) and Shox2 non-V2a (1.3% ± 0.2%) populations (N = 2, 22 sections) (Fig. 3C). Moreover, we also verified that Hb9::Cre-derived INs make up less than 12% ± 2% of the Shox2 non-V2a population (Fig. 3D).

Altogether, these data indicate that although excitatory Hb9::Cre-derived INs are a heterogeneous group of neurons that span several excitatory progenitor domains, they make up a distinct group of excitatory neurons that only marginally overlaps with the Shox2 non-V2a population.

### Vglut2 is effectively removed from excitatory Hb9::Cre-derived INs in Hb9::Cre-Vglut2^Δ/Δ^ mice

In order to selectively target excitatory Hb9::Cre-derived INs and investigate their role in locomotor network activity, *Hb9*::*Cre* mice were crossed with mice carrying a conditional floxed Vglut2 allele to produce offspring with a Cre-dependent selective loss of Vglut2 (see Experimental Procedures), thereby disrupting synaptic transmission exclusively in excitatory Hb9::Cre-derived INs. As shown in previous work from our lab and others, removal of Vglut2 efficiently blocks action potential mediated synaptic transmission from the affected neurons^17^,^26^,^28^, and hence results in the functional removal of these neurons from the network.

**Figure 4:**
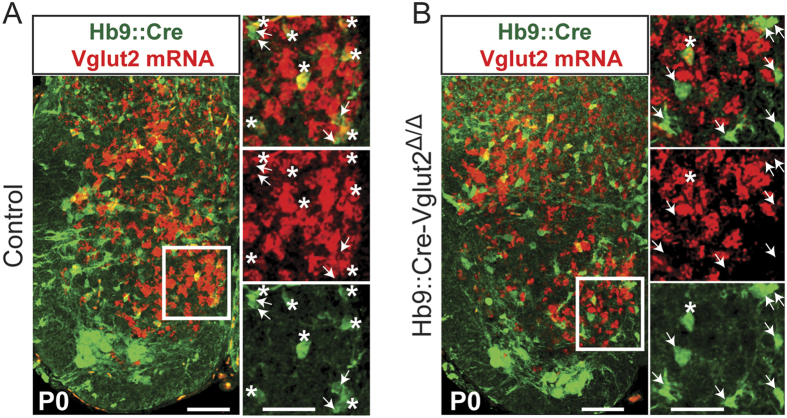
Loss of Vglut2 expression in Hb9::Cre-Vglut2^Δ/Δ^ mice. (**A** and **B**) Fluorescent *in situ* hybridization shows co-localization of *Vglut2* mRNA (red) and Hb9::Cre-derived INs (YFP, green) in control (*Hb9*::*Cre;Vglut2*^*Flox/*+^;*Rosa26-YFP*) (**A**), and Hb9::Cre-Vglut2^Δ/Δ^ (*Hb9*::*Cre;Vglut2*^Δ*/Flox*^;*Rosa26-YFP*) (**B**) P0 spinal cords. Rightmost pictures are magnifications of the white boxed areas. Asterisks indicate *Vglut2* mRNA detected in Hb9::Cre-derived INs. Arrowheads indicate the absence of *Vglut2* mRNA in Hb9::Cre-derived INs. Scale bars represent 100 μm for the transverse hemi-sections and 50 μm for the magnified boxes on the right.

To assess the loss of *Vglut2* transcript, we performed *in situ* hybridization with a Vglut2 probe on spinal cord tissue from both mice which have complete removal of Vglut2 from Hb9::Cre-derived neurons (*Hb9*::*Cre;Vglut2*^Δ*/Flox*^;*Rosa26YFP*, referred to as *Hb9*::*Cre-Vglut2*^Δ*/*Δ^) and littermates which have one copy of Vglut2 removed but retain normal synaptic function (*Hb9*::*Cre;Vglut2*^*Flox/*+^;*Rosa26-YFP*, referred to as controls). Vglut2 signal was seen extensively throughout the gray matter in cords from both control (Fig. 4A) and *Hb9*::*Cre-Vglut2*^Δ*/*Δ^ mice (Fig. 4B). However, *Vglut2* mRNA expression was reduced by 81% (N = 3 animals per condition, 26 sections), in Hb9::Cre-derived INs, in Hb9::Cre-Vglut2^Δ/Δ^ ventral spinal cords compared to controls.

### Silencing the synaptic signaling of excitatory Hb9::Cre-derived INs reduces the frequency of locomotor-like activity

**Figure 5:**
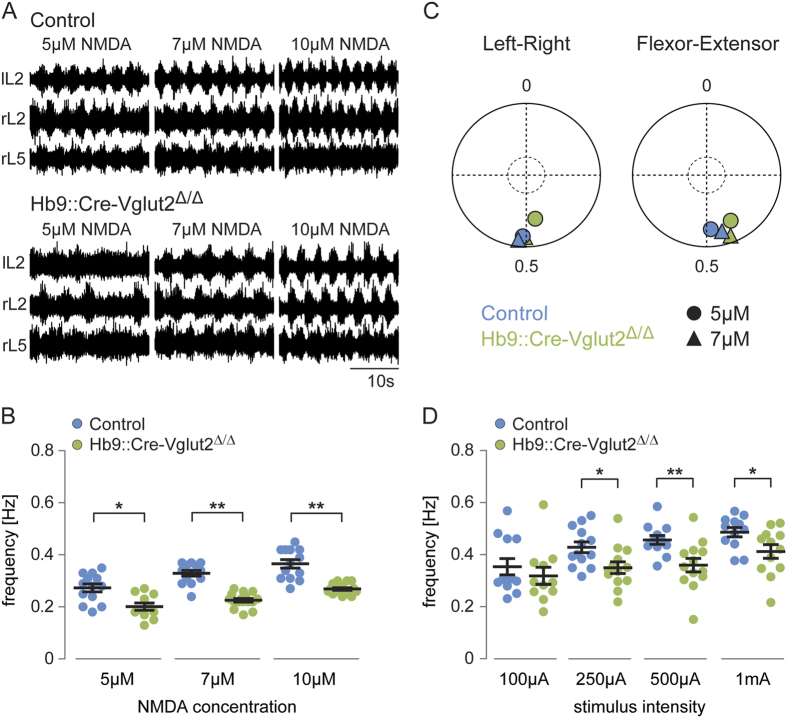
Silencing the output of glutamatergic Hb9::Cre-derived INs leads to reduced frequency of drug-evoked and neural-evoked locomotor-like activity. (**A**) Ventral root recordings of drug-evoked locomotor-like activity in control (upper traces) and Hb9::Cre-Vglut2^Δ/Δ^ (lower traces) spinal cords at three concentrations of NMDA. All include 5-HT (8 μM). (**B**) Frequency of locomotor-like activity as a function of NMDA concentration on a constant background of 5-HT (8 μM) in control (blue) and Hb9::Cre-Vglut2^Δ/Δ^ (green) spinal cords. Locomotor frequencies obtained in Hb9::Cre-Vglut2^Δ/Δ^ cords were always lower than in control cords at all NMDA concentrations. Error bars represent ± SEM. *Indicates p < 0.001 and ** indicates p < 0.0001. (**C**) Circular plots of mean left-right (lL2-rL2) and flexor-extensor (rL2-rL5) coordination in control (blue) and Hb9::Cre-Vglut2^Δ/Δ^ (green) spinal cords at 5 and 7 μM NMDA and 8 μM 5-HT. Each point corresponds to the mean vector value for each drug concentration (Left-right controls: 5 μM and 7 μM NMDA (N = 11); Flexor-extensor controls: 5 μM and 7 μM NMDA (N = 10); Left-right Hb9::Cre-Vglut2^Δ/Δ^ cords: 5 μM NMDA (N = 8) and 7 μM NMDA (N = 13); Flexor-extensor Hb9::Cre-Vglut2^Δ/Δ^ cords: 5 μM NMDA (N = 8) and 7 μM NMDA (N = 12)). Individual vector values were generated from 50 locomotor cycles in each spinal cord. The inner circle indicates significance level of p = 0.05. (**D**) Frequency of the descending fiber-evoked locomotor-like activity as function of stimulation amplitude. The maximal frequency obtained in Hb9::Cre-Vglut2^Δ/Δ^ cords (green) was lower than in controls (blue) at stimulation strengths of 250 μA − 1 mA (controls: 0.35 ± 0.03 Hz (N = 12/13), 0.43 ± 0.02 Hz (N = 13/13), 0.46 ± 0.02 Hz (N = 12/13), 0.49 ± 0.02 Hz (N = 12/13); Hb9::Cre-Vglut2^Δ/Δ^ cords: 0.32 ± 0.03 Hz (N = 11/16), 0.35 ± 0.02 Hz (N = 13/16), 0.36 ± 0.03 Hz (N = 13/16), 0.41 ± 0.03 Hz (N = 12/16) at 100 μA, 250 μA, 500 μA, and 1 mA, respectively). Error bars represent ± SEM. *Indicates p < 0.05 and **Indicates p < 0.005.

To determine the functional impact of the loss of excitatory synaptic transmission in Hb9::Cre-derived INs, we performed locomotor experiments in spinal cords isolated from early postnatal animals. Although *Hb9*::*Cre-Vglut2*^Δ*/*Δ^ mice appear to breathe and have spontaneous movements, they do not survive to P1. Therefore, all experiments were performed at P0. Locomotor-like activity was evoked by bath application of NMDA and serotonin (5-HT). The concentration of 5-HT was kept at 8 μM, while the NMDA was varied (5 μM, 7 μM, and 10 μM) in order to test a range of locomotor frequencies. Locomotor-like activity was evoked in all controls (N = 13/13) (Fig. 5A, upper traces) and Hb9::Cre-Vglut2^Δ/Δ^ cords (5 μM, N = 11/16; 7 μM and 10 μM NMDA, N = 16/16) (Fig. 5A, lower traces). However, locomotor frequencies in Hb9::Cre-Vglut2^Δ/Δ^ cords were 26–31% lower than that of controls at all NMDA concentrations tested (p < 0.0001) (controls: 0.27 ± 0.02 Hz at 5 μM, 0.33 ± 0.01 Hz at 7 μM, 0.36 ± 0.02 Hz at 10 μM; Hb9::Cre-Vglut2^Δ/Δ^ cords: 0.20 ± 0.01 Hz at 5 μM; 0.23 ± 0.006 Hz at 7 μM, 0.27 ± 0.005 Hz at 10 μM) (Fig. 5B). There was no significant difference in pattern generation as assessed by coordination of activity between the left and right sides of the cord (out of phase activity between right L2 and left L2) and in ipsilateral flexor-extensor coordination (out of phase activity between right L2 (flexor) and right L5 (extensor)) between control and Hb9::Cre-Vglut2^Δ/Δ^ cords (Watson-William’s Test, p > 0.09). Mean left-right phase values (Fig. 5C, left) in controls were 0.51 (N = 11) and 0.52 (N = 11) at 5 μM and 7 μM NMDA, respectively, and in Hb9::Cre-Vglut2^Δ/Δ^ cords were 0.47 (5 μM NMDA, N = 8) and 0.50 (7 μM NMDA, N = 13). Mean flexor-extensor phase values (Fig. 5C, right) in controls were 0.49 (5 μM NMDA, N = 10) and 0.46 (7 μM NMDA, N = 10) and in Hb9::Cre-Vglut2^Δ/Δ^ cords were 0.41 (5 μM NMDA, N = 8) and 0.44 (7 μM NMDA, N = 12). Thus, left-right and flexor-extensor bursts were out of phase and close to alternation in both controls and Hb9::Cre-Vglut2^Δ/Δ^ cords.

In addition to drug-evoked locomotion, we also tested the effects of the loss of excitatory Hb9::Cre-derived INs on locomotion evoked by neural stimulation. It was possible to evoke locomotor-like activity by stimulating fibers descending from the brainstem in all control cords tested and in ~80% of the Hb9::Cre-Vglut2^Δ/Δ^ cords (N = 13/16). The bursting frequency was reduced in Hb9::Cre-Vglut2^Δ/Δ^ cords by 15–21% compared to controls at stimulation strengths of 250 μA − 1 mA (p < 0.02) (Fig. 5D). Additionally, during electrical stimulation, the bursting was more difficult to discriminate in spinal cords from *Hb9*::*Cre-Vglut2*^Δ*/*Δ^ mice.

Together, the lower drug-evoked and stimulus-evoked locomotor frequencies seen in spinal cords from *Hb9*::*Cre-Vglut2*^Δ*/*Δ^ mice compared to controls suggest that excitatory spinal Hb9::Cre-derived INs play a significant role in rhythm generation.

### Loss of glutamatergic synaptic transmission in motor neurons does not affect the frequency of drug-induced or neural-evoked locomotor activity

Hb9 is expressed by motor neurons^31^ and mammalian motor neurons co-release glutamate and acetylcholine from central collaterals^17^,^45^,^46^. Since locomotion can be initiated by ventral root stimulation^46^,^47^, we sought to determine if loss of Vglut2-mediated glutamatergic synaptic transmission in motor neurons would contribute to the locomotor phenotype observed in Hb9::Cre-Vglut2^Δ/Δ^ spinal cords.

**Figure 6:**
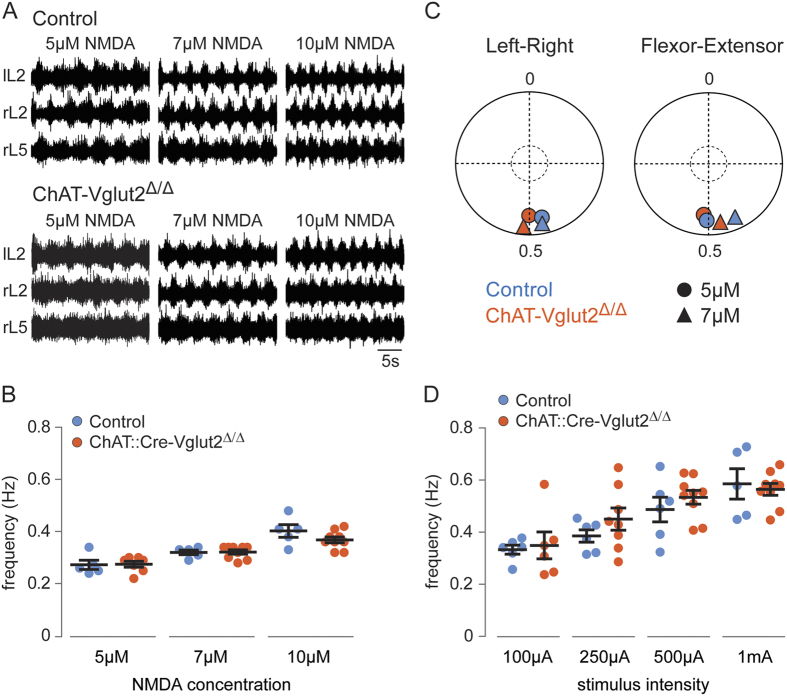
Disruption of glutamatergic synaptic transmission in motor neurons does not affect locomotor frequency. (**A**) Ventral-root recordings showing drug-induced rhythmic locomotor-like activity in control (upper traces) and ChAT-Vglut2^Δ/Δ^ (lower traces) spinal cords at three concentrations of NMDA. All include 5-HT (8 μM). (**B**) Frequency of locomotor-like activity induced by increasing concentrations of NMDA on a constant background of 5-HT (8 μM) in control (blue) and ChAT-Vglut2^Δ/Δ^ (red) spinal cords. Locomotor frequencies obtained in ChAT-Vglut2^Δ/Δ^ cords were similar to that of control cords at all NMDA concentrations. Error bars represent ± SEM. (**C**) Circular plots showing left-right (lL2-rL2) and flexor-extensor (rL2-rL5) phasing in control (blue) and ChAT-Vglut2^Δ/Δ^ (red) spinal cords in different concentrations of NMDA and 8 μM 5-HT. Each point corresponds to the mean vector value of all cords (Left-right controls: 5 μM and 7 μM NMDA (N = 6); Flexor-extensor controls: 5 μM and 7 μM NMDA (N = 4); Left-right ChAT-Vglut2^Δ/Δ^ cords: 5 μM NMDA (N = 8) and 7 μM NMDA (N = 9); Flexor-extensor ChAT-Vglut2^Δ/Δ^ cords: 5 μM NMDA (N = 8) and 7 μM NMDA (N = 9)) for each drug concentration. The inner circle indicates significance level of p = 0.05. (**D**) Frequency of descending fiber-evoked locomotor-like activity as a function of stimulation intensity in controls (blue) and in ChAT-Vglut2^Δ/Δ^ cords (red). Locomotor frequencies obtained in ChAT-Vglut2^Δ/Δ^ cords were similar to that of control cords at all stimulation intensities tested (controls: 0.33 ± 0.02 Hz (N = 6/6), 0.39 ± 0.02 Hz (N = 6/6), 0.49 ± 0.05 Hz (N = 6/6), 0.59 ± 0.06 Hz (N = 5/6); ChAT-Vglut2^Δ/Δ^ cords: 0.35 ± 0.05 Hz (N = 6/9), 0.45 ± 0.04 Hz (N = 8/9), 0.53 ± 0.03 Hz (N = 9/9), 0.56 ± 0.02 Hz (N = 9/9) at 100 μA, 250 μA, 500 μA, and 1 mA, respectively). Error bars represent ± SEM.

Similarly to our approach with *Hb9*::*Cre* mice, *ChAT*::*Cre* mice were used to generate *ChAT-Vglut2*^Δ*/*Δ^ mice in which glutamatergic synaptic transmission was selectively lost in cholinergic neurons, including MNs. *ChAT-Vglut2*^Δ*/*Δ^ mice are viable and show no clear phenotype; however, all experiments were performed at P0 for consistency. Locomotor-like activity was induced by bath application of NMDA (5 μM–10 μM) and 5-HT (8 μM), and it was evoked in all control (N = 5/5) (Fig. 6A, upper traces) and *ChAT-Vglut2*^Δ*/*Δ^ spinal cords (5 μM, N = 7/9; 7 μM and 10 μM NMDA, N = 9/9) (Fig. 6A, lower traces). The locomotor frequency obtained in ChAT-Vglut2^Δ/Δ^ cords was not significantly different from that of controls at any NMDA concentration tested (p > 0.3) (controls: 0.27 ± 0.02 Hz at 5 μM, 0.32 ± 0.01 Hz at 7 μM, 0.40 ± 0.02 Hz at 10 μM; ChAT-Vglut2^Δ/Δ^ cords: 0.27 ± 0.01 Hz at 5 μM; 0.32 ± 0.01 Hz at 7 μM, 0.37 ± 0.01 Hz at 10 μM) (Fig. 6B). Additionally, there was no significant difference in left-right (lL2-rL2) and flexor-extensor (rL2-rL5) phasing between control and ChAT-Vglut2^Δ/Δ^ spinal cords (Watson-William’s Test, p > 0.4). Left-right and flexor-extensor activities were in alternation in both control and ChAT-Vglut2^Δ/Δ^ cords. Mean left-right phase values (Fig. 6C, left) were 0.47 (5 μM, N = 6) and 0.47 (7 μM NMDA, N = 6) for controls, and 0.50 (5 μM NMDA, N = 8) and 0.51 (7 μM NMDA, N = 9) for ChAT-Vglut2^Δ/Δ^ cords. Mean flexor-extensor phase values (Fig. 6C, right) were 0.51 (5 μM, N = 4) and 0.43 (7 μM NMDA, N = 4) for controls, and 0.51 (5 μM NMDA, N = 8) and 0.47 (7 μM NMDA, N = 9) for ChAT-Vglut2^Δ/Δ^ cords.

To further verify if there were any deficits to the rhythm generating network due to the loss of glutamatergic transmission in motor neurons, we also used descending stimulation to evoke locomotion. Locomotor-like activity was evoked in all control (N = 6/6) and ChAT-Vglut2^Δ/Δ^ (N = 9/9) spinal cords. In keeping with the results obtained from drug-induced locomotor studies, upon neural-evoked stimulation, no significant difference in locomotor frequency was observed between ChAT-Vglut2^Δ/Δ^ and control cords at any of the stimulation intensities tested, 100 μA − 1 mA (p > 0.3) (Fig. 6D).

Collectively, these data indicate that the reduction in locomotor frequency we observe in Hb9::Cre-Vglut2^Δ/Δ^ cords is due to the loss of glutamatergic synaptic transmission in Hb9::Cre-derived INs and not in motor neurons.

## Discussion

In this study, we have used the *Hb9*::*Cre* mouse line to identify Hb9::Cre-derived INs, target the excitatory subset of these neurons for removal from the spinal network, and investigate the consequences of their removal on motor output. Our findings provide insight into the involvement of these neurons in mammalian locomotor rhythm generation.

*Hb9*::*Cre* mice^40^ were generated with an analogous strategy to the *Hb9-LacZ* mice^31^. However, a larger population of ventral as well as dorsal neurons is apparent in the *Hb9*::*Cre;Rosa26-FP* mouse. This is possibly due to the transient embryonic expression of Hb9 in progenitor cells and postmitotic neurons^32^ which is captured by the Cre-line. Here, we have collectively referred to the dorsal and ventral populations visualized in *Hb9*::*Cre;Rosa26-FP* mice as Hb9::Cre-derived INs.

Since in limbed animals the spinal locomotor networks are distributed rostro-caudally along the ventral lumbar spinal cord^5^,^20^, the fact that the *Hb9*::*Cre* mouse also includes cells in the dorsal spinal cord does not hinder its use as a tool to study the role of excitatory Hb9::Cre-derived INs in locomotion. Moreover, despite the large number and laminar distribution of Hb9::Cre-derived INs, they comprise a reliably identifiable population of interneurons. The same neurons are visualized by reporter expression in all Rosa26 lines throughout the lumbar spinal cord. Additionally, as demonstrated by *Vglut2* mRNA *in situ*, this same visualized population is manipulated in the *Hb9*::*Cre-Vglut2*^Δ*/*Δ^ mice.

Therefore, by using Cre-Lox recombination, we were able to reliably manipulate a group of excitatory INs which does not correspond to any of the known major excitatory ipsilaterally-projecting classes of neurons. In particular, excitatory Hb9::Cre-derived INs delineate a group of neurons that is distinct from the only other excitatory neuronal population that has been shown to play a role in mammalian locomotor rhythm generation, the Shox2 non-V2a neurons^28^.

The cardinal feature that should characterize rhythm-generating neurons is that their selective manipulation should have a direct impact on locomotor frequency. Their activation should be able to initiate locomotor rhythm and/or change the frequency of the ongoing rhythm whereas a selective reduction in their number should reduce the frequency of the ongoing locomotor rhythm^1^,^4^,^9^,^23^,^28^,^48^,^49^. The most conclusive test to determine the effects of a given group of neurons on locomotor activity is achieved by an acute activation or inactivation of these neurons. However, the combination of channelrhodopsin (or other cell activator or inactivator) mouse lines with *Hb9*::*Cre mice* would lead to expression in both inhibitory and excitatory Hb9::Cre-derived INs as well as in MNs, thus confounding results. The inclusion of MNs in the population makes optogenetic or chemogenetic perturbation experiments impossible to perform. Therefore, we instead pursued the functional removal of glutamatergic Hb9::Cre-derived INs from the network by selectively deleting Vglut2 from the excitatory Hb9::Cre-derived population. By silencing the output of glutamatergic Hb9::Cre-derived INs and studying motor output in an isolated spinal cord preparation, we have shown that excitatory Hb9::Cre-derived INs are likely part of the locomotor rhythm generator, as demonstrated by the reduced locomotor frequency. In contrast, silencing the output of Vglut2-expressing Hb9::Cre-derived INs had no effect on patterning, including left-right and flexor-extensor alternations, unlike what has been described for the manipulation of excitatory V2a, V0 or V3 neurons^12^,^13^,^14^,^16^,^50^. We acknowledge, however, that distinguishing between a role in tonic drive to the rhythm generator and a role in rhythm generation per se is difficult and nearly impossible experimentally, and therefore, we cannot exclude that this decrease in frequency might be partly due to a reduction in tonic drive to the rhythm generating core.

The fact that some of the spinal cords from *Hb9*::*Cre-Vglut2*^Δ*/*Δ^ mice did not respond to descending fiber stimulation may suggest that glutamatergic Hb9::Cre-derived INs play a role in mediating the initiation of locomotion. Thus, it is possible that the descending command system which initiates locomotion may target glutamatergic Hb9::Cre-derived INs and, due to the functional inactivation of these glutamatergic interneurons in *Hb9*::*Cre-Vglut2*^Δ*/*Δ^ mice, locomotion is less readily evoked by stimulation of the descending fibers.

With the genetic tools at hand, it is not possible to determine whether the canonical Hb9 INs are partly or solely accountable for the locomotor phenotype we observe in Hb9::Cre-Vglut2^Δ/Δ^ spinal cords. However, canonical Hb9 INs make up less than 1% of the Hb9::Cre-derived INs, and 33% of these Hb9::Cre-derived INs are excitatory. Therefore, the canonical Hb9 INs would be a negligible portion of the excitatory Hb9::Cre-derived INs (<3%). Accordingly, in Hb9::Cre-Vglut2^Δ/Δ^ cords, the small number of canonical Hb9 neurons as compared to the total number of excitatory Hb9::Cre-derived INs, makes it less likely that the canonical Hb9 interneurons alone are responsible for the observed phenotype.

It should be emphasized that, similarly to what was concluded in studies on Shox2 neurons^28^, we believe that it is unlikely that excitatory Hb9::Cre-derived INs are the sole rhythm-generating neurons in the mammalian locomotor network. In the absence of excitatory Hb9::Cre-derived INs, locomotor frequency is reduced but locomotor rhythm is not abolished. This is seen more clearly when compared to experiments where all excitatory neurons are silenced, which leads to a complete cessation of the rhythm^23^. These observations suggest that several molecularly distinct groups of neurons may contribute to rhythm generation. A distribution of rhythm generation between molecularly distinct groups of excitatory neurons is also seen in young zebrafish where excitatory commissural neurons^51^,^52^,^53^ and V2a domains are involved in rhythm generation^54^,^55^,^56^. However, the corresponding Shox2 INs and excitatory Hb9::Cre-derived INs have not yet been identified in the zebrafish.

Our classification of neurons as Hb9::Cre-derived INs does not sprout from the initial progenitor domain-defined classes of interneurons^7^. Instead, Hb9::Cre-derived INs, here, are those identified in the *Hb9*::*Cre* mouse line^40^. We have shown that excitatory Hb9::Cre-derived INs are a heterogeneous group of neurons that span several of the transcription factor-defined interneuron classes. Similarly, studies on Shox2 INs have shown that even within Shox2 non-V2a neurons, rhythm generation may be distributed amongst neurons derived from several progenitor domains^28^. Collectively, these data suggest that glutamatergic neurons spanning several classes of the known molecularly defined groups of neurons are responsible for rhythm generation. Therefore, it is plausible that, in the same way as new markers for acetylcholinergic spinal neurons^57^,^58^ and inhibitory neurons^59^,^60^ were identified, a comprehensive transcriptome screening to define a finer-grained molecular code of spinal glutamatergic neurons may define new makers for excitatory neurons that may help to clarify the identity of the functional subgroups involved in mammalian locomotor rhythm generation.

### Experimental Procedures

#### Mouse lines

All experimental procedures followed the guidelines of the Animal Welfare Agency and were approved by the local ethical committee, Stockholms Norra Djurförsöksetisk nämnd. The following transgenic lines were used: *Hb9*::*Cre* (B6;129S-Mnx1tm4(cre)Tmj/J) mice obtained from the Jackson Laboratory see ref. 40; *Vglut2*^*Flox/Flox*^ and *Vglut2*^Δ*/*+^ mice kindly provided by Drs. T Hnasko and R. Palmiter^61^; *Vglut2-GFP* mice generated in our lab by Dr. Borgius using BAC recombination with an analogous strategy to the one described previously^62^ (Supplementary Figure 1); *Hb9-GFP* (B6.Cg-Tg(Hlxb9-GFP)1Tmj/J) mice obtained from the Jackson Laboratory; *GlyT2-GFP* mice kindly provided by Dr. H.U. Zeilhofer^63^; *GAD67-GFP* mice provided by Dr. Y. Yanagawa^64^; *ChAT*::*Cre* (B6;129S6-Chattm2(cre)Lowl/J) mice, *Rosa26-YFP* (Rosa26^FloxedSTOP-YFP^) and *Rosa26-tdTomato* (Rosa26^FloxedSTOP-Tdtomato^) mice obtained from the Jackson Laboratory.

For conditional deletion of Vglut2, *Vglut2*^*Flox/Flox*^ mice with loxP sites flanking exon 2 of the Slc17a6 gene, which encodes for Vglut2, were used (see ref. 17). To avoid germ-line recombination we first crossed either *Hb9*::*Cre* or *ChAT*::*Cre* with Vglut2^Δ/+^ mice to obtain the double transgenic lines *Hb9*::*Cre;Vglut2*^Δ*/*+^ *or ChAT*::*Cre;Vglut2*^Δ*/*+^. These double transgenic lines were then crossed with the homozygous *Vglut2*^*Flox/Flox*^;*Rosa26-YFP* mice to generate *Hb9*::*Cre;Vglut2*^Δ*/Flox*^ or *ChAT*::*Cre;Vglut2*^Δ*/Flox*^ mice (see ref. 28), with conditional removal of Vglut2 and conditional expression of YFP.

For neurotransmitter phenotyping *Vglut2-GFP, Gad67-GFP* or *Glyt2-GFP* mice were crossed with the reporter line *Rosa26-tdTomato* to obtain the double transgenic lines *Vglut2-GFP;Rosa26-tdTomato, Gad67-GFP;Rosa26-tdTomato* or *Glyt2-GFP;Rosa26-tdTomato*. These double transgenic lines were then crossed with *Hb9*::*Cre* mice to generate the triple transgenic lines *Hb9*::*Cre;Rosa26-tdTomato;Vglut2-GFP, Hb9*::*Cre;Rosa26-tdTomato;Gad67-GFP*; or *Hb9*::*Cre;Rosa26-tdTomato;Glyt2-GFP*.

#### *In Situ* Hybridization, Immunohistochemistry and Cell Counts

Spinal cords from embryonic mice at stage E11.5 and lumbar spinal cords from newborn mice at P0 were immersion fixed in 4% paraformaldehyde (2 hours at room temperature for immunohistochemistry or 24 hours at 4 °C for *in situ* hybridization), cryopreserved in 30% sucrose overnight, and stored at −80 °C. Serial transverse sections of embryonic spinal cords and newborn lumbar spinal cords were cryostat sectioned (20 μm) and mounted on Menzel-Glaser SuperFrost slides. All slides were stored at −80 °C. Sections were incubated for 24 hours with one or several of the following primary antibodies: rabbit anti-Shox2 #860 (1:32,000, generated against the peptide CKTTSKNSSIADLR), sheep anti-Chx10 (1:400, Chemicon), mouse anti-Isl1 (40.2D6 and 39.4D5) (1:250, DSHB), rabbit anti-Hb9 (1:16000, DSHB). The specificity of these antibodies has been evaluated and described in previous publications (see refs 12, 28 and 35). Secondary antibodies were obtained from Jackson Immunoresearch or Invitrogen, used at 1:500 and incubated for 2 hours at room temperature.

Combined fluorescent *in situ* hybridization and immunofluorescence labeling was performed as previously described^65^ using an antisense digoxigenin (DIG)-labeled RNA probe for vesicular glutamate transporter 2 (Vglut2) that spans base pairs 540 to 983 (produced by Dr. L. Borgius). Sections were blocked with 0.5% Blocking reagent (PerkinElmer) in TNT (0.1 M Tris-Hcl pH7.5, 0.15 M NaCl, 0.1% (wg/vol) Tween20) and incubated with sheep anti-DIG peroxidase (1:2000, Roche Diagnostics). Signal was detected by tyramide-cyanine 3 (Cy3) amplification (1:50, PerkinElmer).

Slides were mounted in Vectashield reagent (Vector Laboratories), and images were scanned on a LSM510 Confocal Microscope (Zeiss Microsystems) using 10x and 20x objectives. Multiple channels were scanned sequentially to prevent fluorescence bleed through and false-positive signals. A contrast enhancement and noise reduction filter were applied in ImageJ for publication images.

Counts were done manually using a cell-counter plug-in in ImageJ, in 4–11 non-adjacent sections per mice (N = 2–4) in each condition. Cellular counts per hemi-section were averaged per individual animal, and a grand-mean ± standard error of the mean (SEM) was calculated across animals to produce percentage of cell per hemi-section bar-graphs.

#### *In Vitro* Recording of Locomotor-Like Activity

Spinal cords from newborn mice at P0 were dissected in ice-cold low Ca^2+^ Ringer’s solution in mM; (111 NaCl, 3 KCl, 11 glucose, 25 NaHCO_3_, 3.7 MgSO_4_, 1.10 KH_2_PO_4_ and 0.25 CaCl_2_) pH adjusted to 7.4 with 95% O_2_/5% CO_2_. All preparations were transferred to a recording chamber and perfused with normal Ringer’s solution in mM; (111 NaCl, 3 KCl, 11 glucose, 25 NaHCO_3_, 1.3 MgSO_4_, 1.1 KH_2_PO_4_, 2.5 CaCl_2_, pH 7.4, aerated with 95% O_2_/5% CO_2_) at a flow rate of 4–5 ml/min. All recordings were performed at room temperature. Locomotor-like activity was either induced by bath application of N-methyl-D-aspartate (NMDA; 5–10 μM) and serotonin (5-HT; 5–8 μM) or evoked by electric stimulation of descending pathways^66^. Briefly, the electrical stimulation protocol entails a large suction electrode (approximately 100 μm in tip diameter) used to deliver large amplitude (0.1–1 mA), long duration (10 ms) and low frequency (2 Hz) stimuli at the midline over the C1–C2 segments. The total duration of each train of stimuli was 60 seconds.

Recordings were obtained using glass suction electrodes attached to lumbar ventral roots (VRs) L2 or L5 on the left and right sides of the cord. VR signals were AC recorded and band-pass filtered from 100 to 1000 Hz (gain 5–10,000), digitized, and acquired with pCLAMP software (version 10; Molecular Devices).

#### Locomotor analysis

Locomotor frequency (Hz) (defined as the reciprocal of locomotor cycles) was calculated from 3–5 min of activity, taken at least 15 min after the initial burst of drug-induced activity, when locomotor-like activity had stabilized. Locomotor-like activity was analysed using rectified, smoothed (time constant of 0.2 s) and filtered AC signals of VR activity in Spike2 (Cambridge Electronic Design). Statistical significance was determined using two-way ANOVA and Sidak’s multiple comparisons test or multiple T test using the Holm-Sidak method, with alpha = 5.000% (p < 0.05). All p-values were adjusted to account for multiple comparisons. Data are expressed as mean ± standard error of the mean (SEM).

For the analysis of left-right and flexor-extensor coordination, 50 random locomotor cycles were selected. Left (l) L2 VR was chosen as the reference trace while right (r) L2 and rL5 VRs were used as test traces. Circular statistics were used to determine the phase relationship between ipsilateral and contralateral VRs during locomotor-like rhythmic activity as previously described^13^,^17^,^20^.

The onset of a locomotor burst in the reference trace corresponds to the phase value of 0 in the circular plot and the onset of the next burst corresponds to a phase value of 1. For test traces, a phase value of 0.5 corresponds to alternation while a phase value of 1 (or 0) corresponds to synchrony. The mean timing of the 50 phase values was calculated as a vector in the circular plot where the resulting vector length (r) reflects the concentration of phase values around the mean. Rayleigh’s test^67^ was used to determine whether *r* values reached statistical significance (p < 0.05). Points falling inside the inner circle are not significant while those outside the inner circle are significant. Data are expressed as grand mean for each drug concentration. Watson-Williams test^67^ was used to determine significant differences amongst conditions (p < 0.05).

## Additional Information

**How to cite this article**: Caldeira, V. *et al*. Spinal Hb9::Cre-derived excitatory interneurons contribute to rhythm generation in the mouse. *Sci. Rep.* **7**, 41369; doi: 10.1038/srep41369 (2017).

**Publisher's note:** Springer Nature remains neutral with regard to jurisdictional claims in published maps and institutional affiliations.

## Supplementary Material

Supplementary Figure 1

## References

[b1] Grillner S. & Jessell T. M. Measured motion: searching for simplicity in spinal locomotor networks. Current opinion in neurobiology 19, 572–586, doi: 10.1016/j.conb.2009.10.011 (2009). 19896834 PMC2951840

[b2] Kiehn O. Locomotor circuits in the mammalian spinal cord. Annual review of neuroscience 29, 279–306, doi: 10.1146/annurev.neuro.29.051605.112910 (2006). 16776587

[b3] Goulding M. Circuits controlling vertebrate locomotion: moving in a new direction. Nature reviews. Neuroscience 10, 507–518, doi: 10.1038/nrn2608 (2009). 19543221 PMC2847453

[b4] Kiehn O. Decoding the organization of spinal circuits that control locomotion. Nature reviews. Neuroscience 17, 224–38, doi: 10.1038/nrn.2016.9 (2016). 26935168 PMC4844028

[b5] Kiehn O. & Kjaerulff O. Distribution of central pattern generators for rhythmic motor outputs in the spinal cord of limbed vertebrates. Annals of the New York Academy of Sciences 860, 110–129 (1998). 9928306 10.1111/j.1749-6632.1998.tb09043.x

[b6] Goulding M. & Pfaff S. L. Development of circuits that generate simple rhythmic behaviors in vertebrates. Current opinion in neurobiology 15, 14–20, doi: 10.1016/j.conb.2005.01.017 (2005). 15721739

[b7] Jessell T. M. Neuronal specification in the spinal cord: inductive signals and transcriptional codes. Nature reviews. Genetics 1, 20–29, doi: 10.1038/35049541 (2000). 11262869

[b8] Kiehn O. Development and functional organization of spinal locomotor circuits. Current opinion in neurobiology 21, 100–109, doi: 10.1016/j.conb.2010.09.004 (2011). 20889331

[b9] McLean D. L. & Dougherty K. J. Peeling back the layers of locomotor control in the spinal cord. Current opinion in neurobiology 33, 63–70, doi: 10.1016/j.conb.2015.03.001 (2015). 25820136 PMC4523447

[b10] Kiehn O. Decoding the organization of spinal circuits that control locomotion. Nature reviews. Neuroscience 17, 224–238, doi: 10.1038/nrn.2016.9 (2016). 26935168 PMC4844028

[b11] Quinlan K. A. & Kiehn O. Segmental, synaptic actions of commissural interneurons in the mouse spinal cord. The Journal of neuroscience: the official journal of the Society for Neuroscience 27, 6521–6530, doi: 10.1523/JNEUROSCI.1618-07.2007 (2007). 17567813 PMC6672441

[b12] Crone S. A. . Genetic ablation of V2a ipsilateral interneurons disrupts left-right locomotor coordination in mammalian spinal cord. Neuron 60, 70–83, doi: 10.1016/j.neuron.2008.08.009 (2008). 18940589

[b13] Talpalar A. E. . Dual-mode operation of neuronal networks involved in left-right alternation. Nature 500, 85–88, doi: 10.1038/nature12286 (2013). 23812590

[b14] Crone S. A., Zhong G., Harris-Warrick R. & Sharma K. In mice lacking V2a interneurons, gait depends on speed of locomotion. The Journal of neuroscience: the official journal of the Society for Neuroscience 29, 7098–7109, doi: 10.1523/JNEUROSCI.1206-09.2009 (2009). 19474336 PMC2731420

[b15] Lanuza G. M., Gosgnach S., Pierani A., Jessell T. M. & Goulding M. Genetic identification of spinal interneurons that coordinate left-right locomotor activity necessary for walking movements. Neuron 42, 375–386 (2004). 15134635 10.1016/s0896-6273(04)00249-1

[b16] Zhang Y. . V3 spinal neurons establish a robust and balanced locomotor rhythm during walking. Neuron 60, 84–96, doi: 10.1016/j.neuron.2008.09.027 (2008). 18940590 PMC2753604

[b17] Talpalar A. E. . Identification of minimal neuronal networks involved in flexor-extensor alternation in the mammalian spinal cord. Neuron 71, 1071–1084, doi: 10.1016/j.neuron.2011.07.011 (2011). 21943604

[b18] Britz O. . A genetically defined asymmetry underlies the inhibitory control of flexor-extensor locomotor movements. eLife 4, doi: 10.7554/eLife.04718 (2015). PMC4604447 26465208

[b19] Zhang J. . V1 and v2b interneurons secure the alternating flexor-extensor motor activity mice require for limbed locomotion. Neuron 82, 138–150, doi: 10.1016/j.neuron.2014.02.013 (2014). 24698273 PMC4096991

[b20] Kjaerulff O. & Kiehn O. Distribution of networks generating and coordinating locomotor activity in the neonatal rat spinal cord *in vitro*: a lesion study. The Journal of neuroscience: the official journal of the Society for Neuroscience 16, 5777–5794 (1996). 8795632 10.1523/JNEUROSCI.16-18-05777.1996 PMC6578971

[b21] Kiehn O. . Excitatory components of the mammalian locomotor CPG. Brain research reviews 57, 56–63, doi: 10.1016/j.brainresrev.2007.07.002 (2008). 17988744

[b22] Hagglund M., Borgius L., Dougherty K. J. & Kiehn O. Activation of groups of excitatory neurons in the mammalian spinal cord or hindbrain evokes locomotion. Nature neuroscience 13, 246–252, doi: 10.1038/nn.2482 (2010). 20081850

[b23] Hagglund M. . Optogenetic dissection reveals multiple rhythmogenic modules underlying locomotion. Proceedings of the National Academy of Sciences of the United States of America 110, 11589–11594, doi: 10.1073/pnas.1304365110 (2013). 23798384 PMC3710792

[b24] Jordan L. M., Liu J., Hedlund P. B., Akay T. & Pearson K. G. Descending command systems for the initiation of locomotion in mammals. Brain research reviews 57, 183–191, doi: 10.1016/j.brainresrev.2007.07.019 (2008). 17928060

[b25] Kiehn O. . Probing spinal circuits controlling walking in mammals. Biochemical and biophysical research communications 396, 11–18, doi: 10.1016/j.bbrc.2010.02.107 (2010). 20494103

[b26] Bui T. V. . Circuits for grasping: spinal dI3 interneurons mediate cutaneous control of motor behavior. Neuron 78, 191–204, doi: 10.1016/j.neuron.2013.02.007 (2013). 23583114 PMC4535710

[b27] Zhong G. . Electrophysiological characterization of V2a interneurons and their locomotor-related activity in the neonatal mouse spinal cord. The Journal of neuroscience: the official journal of the Society for Neuroscience 30, 170–182, doi: 10.1523/JNEUROSCI.4849-09.2010 (2010). 20053899 PMC2824326

[b28] Dougherty K. J. . Locomotor rhythm generation linked to the output of spinal shox2 excitatory interneurons. Neuron 80, 920–933, doi: 10.1016/j.neuron.2013.08.015 (2013). 24267650

[b29] Al-Mosawie A., Wilson J. M. & Brownstone R. M. Heterogeneity of V2-derived interneurons in the adult mouse spinal cord. The European journal of neuroscience 26, 3003–3015, doi: 10.1111/j.1460-9568.2007.05907.x (2007). 18028108

[b30] Lundfald L. . Phenotype of V2-derived interneurons and their relationship to the axon guidance molecule EphA4 in the developing mouse spinal cord. The European journal of neuroscience 26, 2989–3002, doi: 10.1111/j.1460-9568.2007.05906.x (2007). 18028107

[b31] Arber S. . Requirement for the homeobox gene Hb9 in the consolidation of motor neuron identity. Neuron 23, 659–674 (1999). 10482234 10.1016/s0896-6273(01)80026-x

[b32] Lee S. K., Jurata L. W., Funahashi J., Ruiz E. C. & Pfaff S. L. Analysis of embryonic motoneuron gene regulation: derepression of general activators function in concert with enhancer factors. Development 131, 3295–3306, doi: 10.1242/dev.01179 (2004). 15201216

[b33] Wilson J. M. . Conditional rhythmicity of ventral spinal interneurons defined by expression of the Hb9 homeodomain protein. The Journal of neuroscience: the official journal of the Society for Neuroscience 25, 5710–5719, doi: 10.1523/JNEUROSCI.0274-05.2005 (2005). 15958737 PMC6724883

[b34] Wilson J. M., Cowan A. I. & Brownstone R. M. Heterogeneous electrotonic coupling and synchronization of rhythmic bursting activity in mouse Hb9 interneurons. Journal of neurophysiology 98, 2370–2381, doi: 10.1152/jn.00338.2007 (2007). 17715199

[b35] Hinckley C. A., Hartley R., Wu L., Todd A. & Ziskind-Conhaim L. Locomotor-like rhythms in a genetically distinct cluster of interneurons in the mammalian spinal cord. Journal of neurophysiology 93, 1439–1449, doi: 10.1152/jn.00647.2004 (2005). 15496486

[b36] Hinckley C. A. & Ziskind-Conhaim L. Electrical coupling between locomotor-related excitatory interneurons in the mammalian spinal cord. The Journal of neuroscience: the official journal of the Society for Neuroscience 26, 8477–8483, doi: 10.1523/JNEUROSCI.0395-06.2006 (2006). 16914672 PMC6674344

[b37] Brownstone R. M. & Wilson J. M. Strategies for delineating spinal locomotor rhythm-generating networks and the possible role of Hb9 interneurones in rhythmogenesis. Brain research reviews 57, 64–76, doi: 10.1016/j.brainresrev.2007.06.025 (2008). 17905441 PMC5061561

[b38] Hinckley C. A., Wiesner E. P., Mentis G. Z., Titus D. J. & Ziskind-Conhaim L. Sensory modulation of locomotor-like membrane oscillations in Hb9-expressing interneurons. Journal of neurophysiology 103, 3407–3423, doi: 10.1152/jn.00996.2009 (2010). 20393069 PMC2888255

[b39] Ziskind-Conhaim L., Mentis G. Z., Wiesner E. P. & Titus D. J. Synaptic integration of rhythmogenic neurons in the locomotor circuitry: the case of Hb9 interneurons. Annals of the New York Academy of Sciences 1198, 72–84, doi: 10.1111/j.1749-6632.2010.05533.x (2010). 20536922 PMC3057624

[b40] Yang X. . Patterning of muscle acetylcholine receptor gene expression in the absence of motor innervation. Neuron 30, 399–410 (2001). 11395002 10.1016/s0896-6273(01)00287-2

[b41] Wichterle H., Lieberam I., Porter J. A. & Jessell T. M. Directed differentiation of embryonic stem cells into motor neurons. Cell 110, 385–397 (2002). 12176325 10.1016/s0092-8674(02)00835-8

[b42] Hess D. M. . Localization of TrkC to Schwann cells and effects of neurotrophin-3 signaling at neuromuscular synapses. The Journal of comparative neurology 501, 465–482, doi: 10.1002/cne.21163 (2007). 17278135

[b43] Liem K. F. Jr., Tremml G. & Jessell T. M. A role for the roof plate and its resident TGFbeta-related proteins in neuronal patterning in the dorsal spinal cord. Cell 91, 127–138 (1997). 9335341 10.1016/s0092-8674(01)80015-5

[b44] Helms A. W. & Johnson J. E. Specification of dorsal spinal cord interneurons. Current opinion in neurobiology 13, 42–49 (2003). 12593981 10.1016/s0959-4388(03)00010-2

[b45] Nishimaru H., Restrepo C. E., Ryge J., Yanagawa Y. & Kiehn O. Mammalian motor neurons corelease glutamate and acetylcholine at central synapses. Proceedings of the National Academy of Sciences of the United States of America 102, 5245–5249, doi: 10.1073/pnas.0501331102 (2005). 15781854 PMC555035

[b46] Mentis G. Z. . Noncholinergic excitatory actions of motoneurons in the neonatal mammalian spinal cord. Proceedings of the National Academy of Sciences of the United States of America 102, 7344–7349, doi: 10.1073/pnas.0502788102 (2005). 15883359 PMC1091756

[b47] Pujala A., Blivis D. & O’Donovan M. J. Interactions between Dorsal and Ventral Root Stimulation on the Generation of Locomotor-Like Activity in the Neonatal Mouse Spinal Cord. eNeuro 3, doi: 10.1523/ENEURO.0101-16.2016 (2016). PMC4937207 27419215

[b48] Roberts A., Li W. C. & Soffe S. R. How neurons generate behavior in a hatchling amphibian tadpole: an outline. Frontiers in behavioral neuroscience 4, 16, doi: 10.3389/fnbeh.2010.00016 (2010). 20631854 PMC2903309

[b49] El Manira A. Dynamics and plasticity of spinal locomotor circuits. Current opinion in neurobiology 29, 133–141, doi: 10.1016/j.conb.2014.06.016 (2014). 25062504

[b50] Zhong G., Sharma K. & Harris-Warrick R. M. Frequency-dependent recruitment of V2a interneurons during fictive locomotion in the mouse spinal cord. Nature communications 2, 274, doi: 10.1038/ncomms1276 (2011). PMC3597081 21505430

[b51] McLean D. L., Masino M. A., Koh I. Y., Lindquist W. B. & Fetcho J. R. Continuous shifts in the active set of spinal interneurons during changes in locomotor speed. Nature neuroscience 11, 1419–1429, doi: 10.1038/nn.2225 (2008). 18997790 PMC2735137

[b52] Fetcho J. R. & McLean D. L. Some principles of organization of spinal neurons underlying locomotion in zebrafish and their implications. Ann Ny Acad Sci 1198, 94–104, doi: 10.1111/j.1749-6632.2010.05539.x (2010). 20536924 PMC3579554

[b53] McLean D. L. & Fetcho J. R. Spinal Interneurons Differentiate Sequentially from Those Driving the Fastest Swimming Movements in Larval Zebrafish to Those Driving the Slowest Ones. Journal of Neuroscience 29, 13566–13577, doi: 10.1523/Jneurosci.3277-09.2009 (2009). 19864569 PMC2796107

[b54] McLean D. L. & Fetcho J. R. Using imaging and genetics in zebrafish to study developing spinal circuits *in vivo*. Developmental neurobiology 68, 817–834, doi: 10.1002/dneu.20617 (2008). 18383546 PMC3579555

[b55] Eklof-Ljunggren E. . Origin of excitation underlying locomotion in the spinal circuit of zebrafish. Proceedings of the National Academy of Sciences of the United States of America 109, 5511–5516, doi: 10.1073/pnas.1115377109 (2012). 22431619 PMC3325722

[b56] Ljunggren E. E., Haupt S., Ausborn J., Ampatzis K. & El Manira A. Optogenetic activation of excitatory premotor interneurons is sufficient to generate coordinated locomotor activity in larval zebrafish. The Journal of neuroscience: the official journal of the Society for Neuroscience 34, 134–139, doi: 10.1523/JNEUROSCI.4087-13.2014 (2014). 24381274 PMC6608174

[b57] Enjin A. . Identification of novel spinal cholinergic genetic subtypes disclose Chodl and Pitx2 as markers for fast motor neurons and partition cells. The Journal of comparative neurology 518, 2284–2304, doi: 10.1002/cne.22332 (2010). 20437528

[b58] Zagoraiou L. . A cluster of cholinergic premotor interneurons modulates mouse locomotor activity. Neuron 64, 645–662, doi: 10.1016/j.neuron.2009.10.017 (2009). 20005822 PMC2891428

[b59] Bikoff J. B. . Spinal Inhibitory Interneuron Diversity Delineates Variant Motor Microcircuits. Cell, doi: 10.1016/j.cell.2016.01.027 (2016). PMC4808435 26949184

[b60] Perry S. . Firing properties of Renshaw cells defined by Chrna2 are modulated by hyperpolarizing and small conductance ion currents Ih and ISK. The European journal of neuroscience 41, 889–900, doi: 10.1111/ejn.12852 (2015). 25712471

[b61] Hnasko T. S. . Vesicular glutamate transport promotes dopamine storage and glutamate corelease *in vivo*. Neuron 65, 643–656, doi: 10.1016/j.neuron.2010.02.012 (2010). 20223200 PMC2846457

[b62] Borgius L., Restrepo C. E., Leao R. N., Saleh N. & Kiehn O. A transgenic mouse line for molecular genetic analysis of excitatory glutamatergic neurons. Molecular and cellular neurosciences 45, 245–257, doi: 10.1016/j.mcn.2010.06.016 (2010). 20600924

[b63] Zeilhofer H. U. . Glycinergic neurons expressing enhanced green fluorescent protein in bacterial artificial chromosome transgenic mice. The Journal of comparative neurology 482, 123–141, doi: 10.1002/cne.20349 (2005). 15611994

[b64] Tamamaki N. . Green fluorescent protein expression and colocalization with calretinin, parvalbumin, and somatostatin in the GAD67-GFP knock-in mouse. The Journal of comparative neurology 467, 60–79, doi: 10.1002/cne.10905 (2003). 14574680

[b65] Borgius L. . Spinal Glutamatergic Neurons Defined by EphA4 Signaling Are Essential Components of Normal Locomotor Circuits. The Journal of neuroscience: the official journal of the Society for Neuroscience 34, 3841–3853, doi: 10.1523/JNEUROSCI.4992-13.2014 (2014). 24623763 PMC6705281

[b66] Talpalar A. E. & Kiehn O. Glutamatergic mechanisms for speed control and network operation in the rodent locomotor CpG. Frontiers in neural circuits 4, doi: 10.3389/fncir.2010.00019 (2010). PMC2938926 20844601

[b67] Zar J. H. Biostatistical analysis. (Prentice-Hall, 1974).

