# Supplementary material for: Spinal Hb9::Cre-derived excitatory interneurons contribute to rhythm generation in the mouse
Source: Sci Rep. 2017 Jan 27;7:41369. doi: 10.1038/srep41369 (PMC5269678; doi:10.1038/srep41369)
Supplement: Supplementary Figure 1 [file srep41369-s1.pdf]

Supplementary Material

Spinal Hb9::Cre-derived excitatory interneurons contribute to rhythm generation in the mouse

Vanessa Caldeira[1], Kimberly J. Dougherty[1,2,*], Lotta Borgius[1] and Ole Kiehn[1,*]

[1]Mammalian Locomotor Laboratory, Department of Neuroscience, Karolinska Institutet, 171 77 Stockholm, Sweden, [2]Department of Neurobiology and Anatomy, Drexel University College of Medicine, Philadelphia, PA 19129, US

Supplementary Figure 1. Characterization of Vglut2-GFP mice. *Specificity of transgene expression in Vglut2-GFP mice was assessed by crosses with the well described Vglut2::Cre mouse* [1].

(A) Co-expression of GFP (green) and Cre antibody (red) in the *Vglut2-GFP;Vglut2::Cre* mouse spinal cord at P0. Rightmost pictures are magnifications of the white boxed area and indicate overlap between Vglut2-GFP (green) and Vglut2::Cre (red) INs. Scale bars: 100 µm.

(B) Bar graph showing the percent overlap between GFP^+ and Cre ab^+ (Cre antibody positive) cells in *Vglut2-GFP;Vglut2::Cre* mice. The majority of Vglut2-GFP cells also co-express Cre antibody (96% ± 1%) (N = 3, 12 sections).

1 Borgius, L., Restrepo, C. E., Leao, R. N., Saleh, N. & Kiehn, O. A transgenic mouse line for molecular genetic analysis of excitatory glutamatergic neurons. *Molecular and cellular neurosciences* **45**, 245-257, doi:10.1016/j.mcn.2010.06.016 (2010).

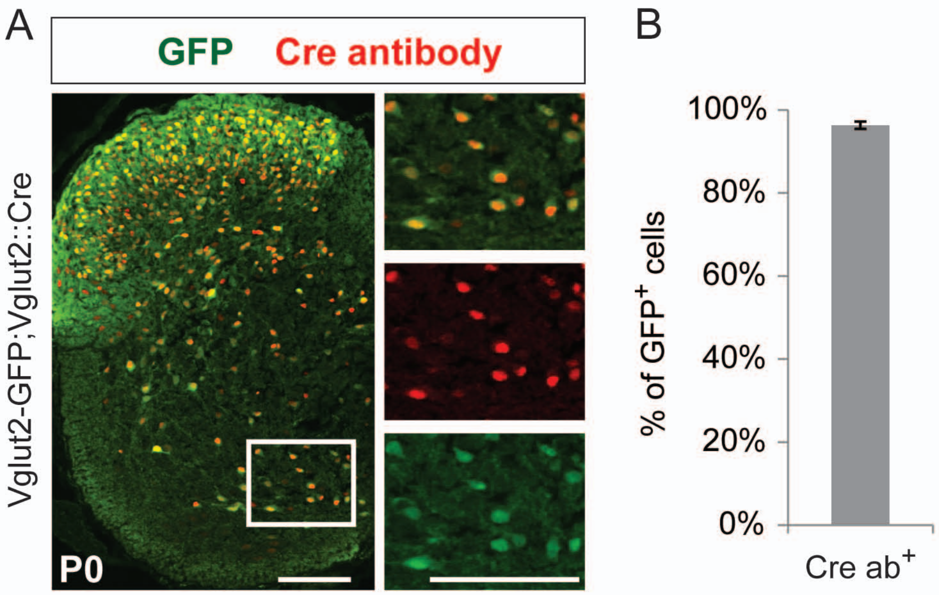
A
GFP
Cre antibody
Vglut2-GFP;Vglut2::Cre
P0
B
100%
80%
60%
40%
20%
0%
% of GFP+ cells
Cre ab+
